# Concordant clear cell “mesonephric” carcinoma of the bladder and lung adenocarcinoma with clear cell features – multiple primaries versus metastatic neoplasms: a case report 

**DOI:** 10.1186/s13256-017-1295-2

**Published:** 2017-05-12

**Authors:** Sarmad H. Jassim, Amer Khiyami, Jane K. Nguyen, Santhi Ganesan, Joseph Tomashefski, Joram Sawady

**Affiliations:** Department of Pathology, MetroHealth Medical Center, Case Western Reserve University, Cleveland, OH USA

**Keywords:** Lung cancer, Bladder cancer, Clear cells, Adenocarcinoma, *ARID2*, TTF-1, Case report

## Abstract

**Background:**

Clear cell carcinoma of the bladder is a rare variant of urinary bladder adenocarcinoma. We report a case of a patient with clear cell carcinoma of the bladder and a concordant right upper lobe pulmonary adenocarcinoma with clear cell features, and we address the role of immunohistochemistry and cytogenetic analysis in distinguishing the two primary malignancies.

**Case presentation:**

Our patient was a 59-year-old African American woman who presented with hematuria. Her past medical history included invasive mammary carcinoma and end-stage renal disease treated with hemodialysis. A computed tomographic urogram revealed a 3-cm polypoid bladder mass. A follow-up chest computed tomographic scan revealed a 1-cm right upper lobe nodule. The patient underwent transurethral biopsy and subsequent radical cystectomy, as well as a transthoracic core needle biopsy of the lung nodule. Histologically, the bladder tumor consisted of flat, cuboidal to columnar cells with clear or eosinophilic cytoplasm and a hobnail appearance, organized in tubulocystic and papillary patterns. The neoplastic cells were diffusely positive for α-methylacyl-coenzyme A racemase, cancer antigen 125, and cytokeratin 7; focally positive for cytokeratin 20, P53, and carcinoembryonic antigen; and negative for thyroid transcription factor 1. The lung tumor demonstrated a glandular architecture with mucin production (positive for mucin with mucicarmine and periodic acid-Schiff with diastase stain). The neoplastic cells were diffusely positive for cytokeratin 7, napsin A, and thyroid transcription factor 1, and they were negative for cytokeratin 20 and cancer antigen 125. Genetic testing of the pulmonary neoplasm demonstrated *ARID2* genomic alterations.

**Conclusions:**

The presence of clear cell features in both neoplasms raised the possibility of lung metastasis from the primary bladder tumor. However, the glandular architecture of the lung neoplasm along with its distinctive immunohistochemical and genetic profiles confirmed the presence of two separate primaries.

## Background

Clear cell carcinoma of the bladder (CCCB) is a rare variant of urinary bladder carcinoma [1–4]. It is more frequent in females and has a wide age distribution [1]. CCCB should be differentiated from urothelial carcinoma with clear cell features, which is more common in males [1]. CCCB was first reported in 1968 by Dow and Young [2] and was designated as mesonephric carcinoma (MC) because it was suspected to be of mesonephric origin [2–9]. Nine years later, in a second published case report, Skor and Warren elaborated on the histogenesis of CCCB or MC [3]. Those authors hypothesized that the neoplasm originated from metaplasia of the urothelium and anaplasia of embryonic cell rests [3]. Currently, there is no convincing evidence for a mesonephric origin [1, 9]. In the most recent 2016 World Health Organization (WHO) classification, CCCB is classified under tumors of Müllerian type, which arise from existing Müllerian precursors within the urinary bladder, commonly endometriosis and rarely Müllerianosis [1].

Patients with CCCB commonly present with hematuria, urinary urgency and frequency, dysuria, urinary retention, and/or a history of repeated urinary tract infections [1, 4, 9]. The gross appearance is nonspecific, but the tumor frequently grows as a papillary mass [1, 8–10]. The most common locations are the bladder neck, trigone, and lateral bladder wall [1, 4, 6, 9, 11] and the dome [12]. The most common architectural pattern is tubulocystic, followed by papillary, glandular, and diffuse [1, 8–13]. The tumor cells range from flat to cuboidal or columnar and may have clear or eosinophilic cytoplasm, or an admixture of both with prominent hobnailing [1, 10–13]. Clear cell appearance can be seen in a wide variety of carcinomas arising from different sites, including prostate, breast, uterus, ovary, vagina, lung, and kidney. Hence, the differential diagnosis of CCCB includes urothelial carcinoma with clear cells [1], nephrogenic adenoma [7, 9, 13], metastatic clear cell renal cell carcinoma [14], and cervical or vaginal clear cell adenocarcinoma [1], among others. Patients with renal cell carcinoma frequently present with metastasis, including bladder metastasis [14]. Immunohistochemical studies have shown that CCCB is positive for cytokeratin 7 (CK7), CAM5.2 (a low-molecular-weight keratin), epithelial membrane antigen, paired box 8 (PAX8), hepatocyte nuclear factor 1β, α-methylacyl-coenzyme A racemase (AMACR), and cancer antigen 125 (CA-125) [1]. It may also be positive for cluster of differentiation 10, uroplakin, CK20, Lewis X antigen, PAX2, and carcinoembryonic antigen (CEA) [1, 10, 15]. It is negative for prostate-specific antigen, prostate-specific acid phosphatase, protein 63, 34ßE12 (a cytokeratin high-molecular-weight antibody), estrogen and progesterone receptors, and GATA-binding protein 3 [1, 15]. The presence of a prominent clear cell population, significant pleomorphism and cytological atypia, brisk mitotic activity, and protein 53 (P53) staining with high methylation inhibited binding protein-1 (MIB-1) activity favor the diagnosis of CCCB [1, 9, 13, 15]. These carcinomas display chromosomal alterations similar to those of urothelial carcinomas, including gains on chromosomes 3, 7, and 17 and X chromosome inactivation [1]. Clinically, CCCB may not be as aggressive as initially believed. Exophytic tumors that are diagnosed early and completely removed carry a relatively better prognosis [1]. Recurrence after limited surgical resection is not uncommon and has been reported [16].

According to the WHO 2015 lung tumor classification, clear cells in pulmonary adenocarcinoma are considered a component of the solid, acinar, papillary, and micropapillary adenocarcinoma patterns in decreasing frequency, and not a primary histological subtype of lung adenocarcinoma [17]. The WHO therefore recommends that these neoplasms be reported as pulmonary adenocarcinomas with clear cell features [17].

In this article, we report a case of a patient with clear cell adenocarcinoma (MC) of the urinary bladder and urethra with a concordant right upper lobe pulmonary adenocarcinoma with clear cell features, and we address the important immunohistochemical distinction between the two primary tumors. In addition, we present the pathological and clinical challenges of diagnosing multiple primaries versus metastatic neoplasms in our patient.

## Case presentation

A 59-year-old African American woman who was a former smoker presented to our hospital with a 3-month history of worsening urinary symptoms, including voiding difficulties and one episode of gross hematuria. Her past medical history was significant for hypertension, type 2 diabetes mellitus, hyperlipidemia, cerebrovascular disease with residual right hemiparesis, and end-stage renal disease requiring hemodialysis. She also underwent remote left adrenal adenoma excision and was remotely treated for invasive mammary carcinoma by surgery and adjuvant radiotherapy. The patient declined chemotherapy. The patient had a smoking history of 30 pack-years, but she had quit smoking approximately 20 years ago after receiving a diagnosis of cerebrovascular disease. A review of her social and environmental history did not indicate exposure to any other known toxins or carcinogens. The patient denied any family history of cancer and reported that both her parents had died of “old age.” The patient is living with her husband, who is her primary caregiver and provides physical and social support.

The patient’s medication list at this presentation included metoprolol (25 mg) for hypertension, calcium acetate (667 mg three times daily) for renal failure, and simvastatin (40 mg) for hyperlipidemia. The patient was also taking omeprazole (40 mg) for gastroesophageal reflux disease, polyethylene glycol for constipation, paroxetine (30 mg) for anxiety and depression, and latanoprost 0.0005% eye drops for glaucoma. Her diabetes was under dietary control.

A review of systems was negative for nausea, vomiting, loss of appetite, and diarrhea. The patient denied cough, chest pain, and shortness of breath. The patient’s vital signs at this presentation were stable, with a blood pressure of 110/40 mmHg, pulse of 65 beats/minute, and oral temperature of 98.4 °F. The patient was well nourished, oriented, and used a wheelchair for mobilization. Her physical examination revealed a well-healed surgical scar over her right breast and hemodialysis scars on her upper extremities. No palpable lymph nodes were detected. Her lungs were clear to auscultation bilaterally, with no wheezing, rales, or rhonchi. Her abdomen was soft and nontender with positive bowel sounds and no hepatosplenomegaly. Her neurological examination revealed residual right hemiparesis and slurred speech. Urinalysis confirmed microscopic hematuria with a large amount of blood. Her complete blood count revealed a white blood cell count of 6900/μl, hemoglobin of 12.4 g/dl, red blood cell count of 4.01 × 10^6^/μl, hematocrit of 38.7%, and platelet count of 198,000/μl. The patient’s liver function tests revealed total protein of 6.0 g/dl, albumin of 3.3 g/dl, direct bilirubin of 0.6 mg/dl, and total bilirubin of 1.7 mg/dl, with normal alkaline phosphatase, aspartate aminotransferase, and alanine aminotransferase at 65 IU/L, 11 IU/L, and 21 IU/L, respectively. The patient’s basic metabolic panel was maintained by continuous need for hemodialysis three times per week, given her history of end-stage renal disease. A urine culture yielded more than 10,000 colony-forming units per milliliter of *Escherichia coli*, which was treated conservatively.

**Fig. 1:**
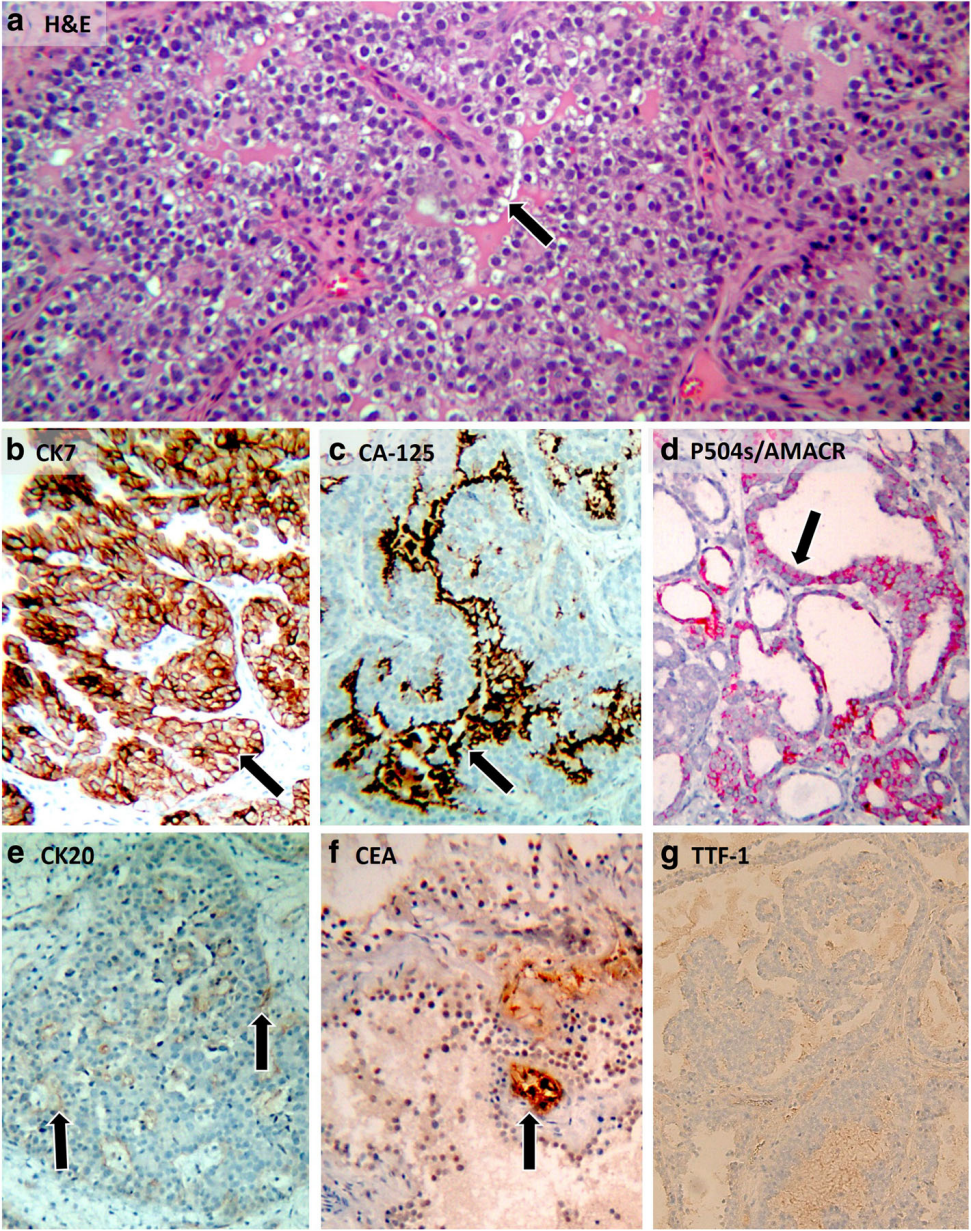
Clear cell “mesonephric” carcinoma of the bladder. **a** Hematoxylin and eosin stain demonstrating characteristic clear cell appearance of the neoplastic cells (*arrow*). **b** Cytokeratin 7 staining diffusely positive in neoplastic cells (*arrow*). **c** Cancer antigen 125-positive in neoplastic cells (*arrow*). **d** P504S/α-methylacyl-coenzyme A racemase is positive in neoplastic cells (*arrow*). **e** Cytokeratin 20 is only focally positive in neoplastic cells (*arrows*). **f** Carcinoembryonic antigen is focally positive in neoplastic cells (*arrow*). **g** Thyroid transcription factor 1 staining is negative. *AMACR* α-Methylacyl-coenzyme A racemase, *CA-125* Cancer antigen 125, *CEA* Carcinoembryonic antigen, *CK* Cytokeratin, *H&E* Hematoxylin and eosin, *P53* Protein 53, *TTF-1* Thyroid transcription factor 1

A computed tomographic (CT) urogram revealed a 3-cm polypoid bladder mass involving the posterior inferior bladder wall near the neck. The patient underwent transurethral resection of bladder tumor. Histologically, the hematoxylin and eosin-stained slides of the bladder tumor showed flat, cuboidal to columnar cells with clear to eosinophilic cytoplasm and a hobnail appearance, organized in tubulocystic and papillary patterns (Fig. 1a). The neoplastic cells were diffusely positive for CK7, CA-125, and AMACR; focally positive for CK20, P53, and CEA; and negative for thyroid transcription factor 1 (TTF-1) (Fig. 1b–g and Table 1). A diagnosis of stage T3N0Mx clear cell (mesonephric) carcinoma of the urinary bladder was made.

**Table 1:** Immunohistochemical profiles of the two neoplasms

	CK7	CK20	CEA	CA-125	AMACR	P53	TTF-1	Napsin A	PAS-D/mucicarmine
TURBT (CCCB)	Diffusely positive	Focally positive	Focally positive	Positive	Positive	Focally positive	Negative	ND	ND
Lung nodule	Diffusely positive	Negative	ND	Negative	ND	ND	Positive	Positive	Positive

*Abbreviations: AMACR* α-Methylacyl-coenzyme A racemase, *CA-125* Cancer antigen 125, *CCCB* Clear cell carcinoma of the bladder, *CEA* Carcinoembryonic antigen, *CK* Cytokeratin, *ND* Not done, *P53* Protein 53, *PAS-D* periodic acid-Schiff with diastase stain, *TTF-1* Thyroid transcription factor 1, *TURBT* Transurethral resection of bladder tumor

The immunohistochemical profiles of the CCCB and the lung adenocarcinoma with clear cell features are displayed

**Fig. 2:**
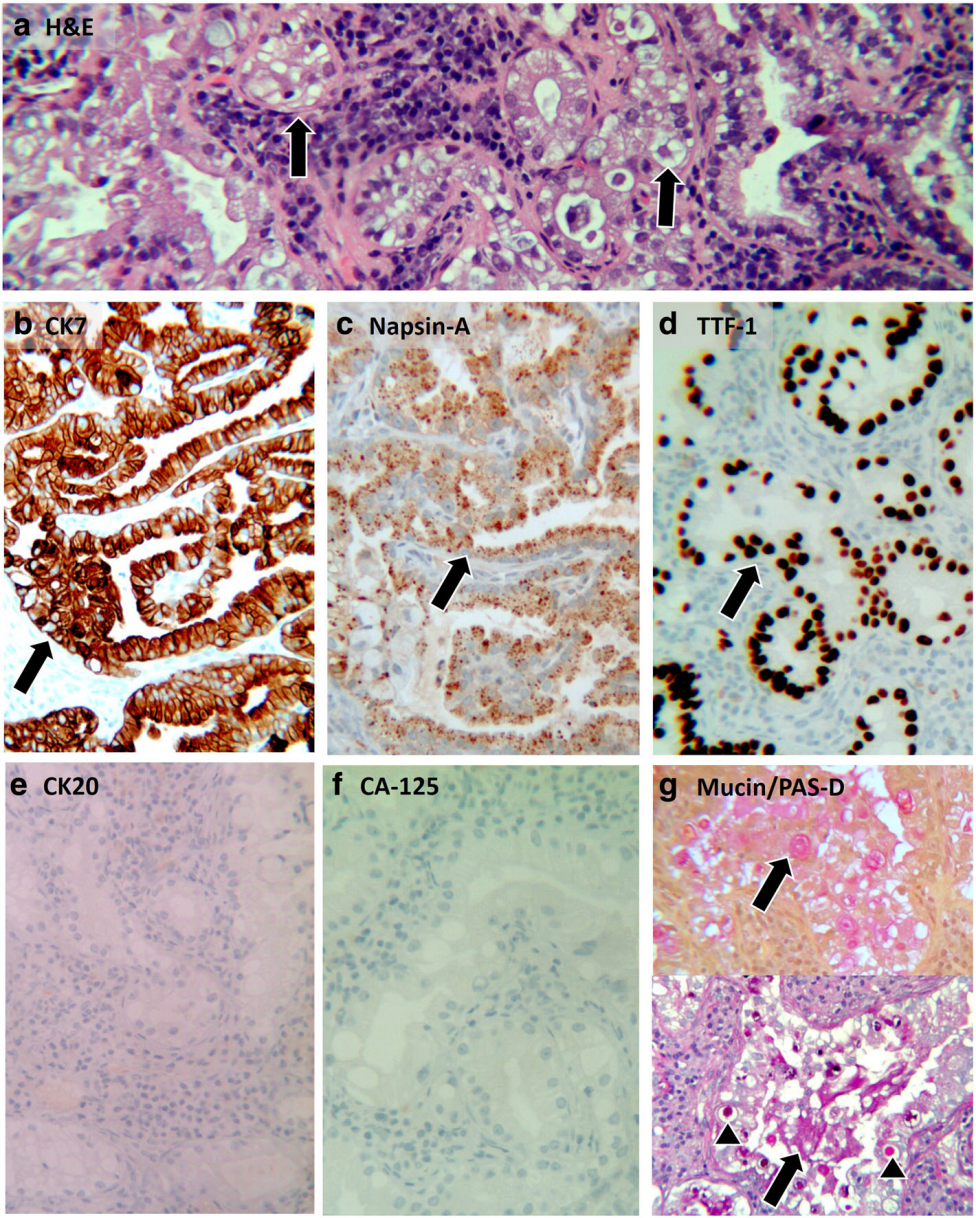
Lung adenocarcinoma with clear cell features. **a** Hematoxylin and eosin stain demonstrating characteristic glandular architecture of the neoplastic cells with clearing of the cytoplasm (*arrows*). **b** Cytokeratin 7 staining diffusely positive in neoplastic cells (*arrow*). **c** Napsin A-positive staining in neoplastic cells (*arrow*). **d** Thyroid transcription factor 1 staining is positive in neoplastic cells (*arrow*). **e** Cytokeratin 20 staining is negative in neoplastic cells. **f** Cancer antigen 125 staining is negative in neoplastic cells. **g** Mucin and periodic acid-Schiff with diastase staining are positive in neoplastic cells (both extracellular [*arrow*] and intracellular [*arrowheads*]). *AMACR* α-Methylacyl-coenzyme A racemase, *CA-125* Cancer antigen 125, *CEA* Carcinoembryonic antigen, *CK* Cytokeratin, *H&E* Hematoxylin and eosin, *P53* Protein 53, *PAS-D* Periodic acid-Schiff with diastase stain, *TTF-1* Thyroid transcription factor 1

Subsequently, after the diagnosis of the CCCB, a staging chest CT scan revealed a 6.4×7.1-mm nodule in the right upper lobe. The patient did not complain of respiratory symptoms at the time the mass was detected. This was followed by a transthoracic core needle biopsy. On the basis of histology, the pulmonary nodule demonstrated a glandular architecture with mucin production (positive staining with mucicarmine and periodic acid-Schiff with diastase) (Fig. 2a, g, and Table 1). The neoplastic cells were diffusely positive for CK7, napsin A, and TTF-1 and negative for CK20 and CA-125 (Fig. 2b–f and Table 1). Genetic testing revealed *ARID2* and *KEAP1* genomic alterations. A diagnosis of mucin-producing adenocarcinoma with clear cell features, consistent with lung primary, was rendered on the basis of the morphological, immunohistochemical, and genetic profile of the tumor.

The patient subsequently underwent a radical cystectomy (RC) with bilateral salpingo-oophorectomy and hysterectomy. A tumor measuring 3.5×2.0×1.7 cm was noted within the bladder neck and appeared to be extending into the upper urethra. The tumor was tannish gray, firm, and homogeneous. Microscopically, the invasive clear cell (mesonephric) carcinoma extended focally into the perivesicular tissue. The remaining parts of the specimen were negative for malignancy. The patient was treated with adjuvant radiotherapy in two 6000-cGy doses in 20 and 15 fractions each, 18 months apart, as hypofractionation radiation cycles aimed at the lung nodule. The patients’ medications were adjusted accordingly to metoprolol (XL 50 mg), amlodipine (10 mg) and aspirin (81 mg) were added for hypertension, and bisacodyl replaced her previous medication for constipation. She had multiple complex medical and surgical presentations and received a multidisciplinary departmental approach in her management. She is doing well 3 years following the diagnosis of CCCB. The pulmonary nodule was radiologically stable in size on the most recent follow-up CT scan.

## Discussion

To the best of our knowledge, this is the first report that describes a patient with CCCB with a concordant pulmonary adenocarcinoma with clear cell features. Although concurrent bladder and lung cancers are recognized today more frequently than before, with the bladder cancer being mostly the first primary detected, all the reported bladder cancers were transitional cell carcinomas (urothelial carcinoma) [18]. The possibility of a bladder metastasis from a renal [19] or ovarian clear cell carcinoma [20] was excluded in our patient because imaging did not reveal other genitourinary masses. In addition, the RC specimen confirmed the bladder neoplasm location and did not reveal other genital neoplasms. The presence of clear cell features in both neoplasms in our patient raised the possibility of metastasis from one organ to the other. The lung neoplasm, however, was morphologically dissimilar from the bladder tumor. The glandular architecture with intracellular and extracellular mucin production (Fig. 2a, g, and Table 1) militates against a pulmonary metastasis from the bladder because the latter is not known to produce mucin, but it is known to produce glycogen [20] and is usually described as tubulocystic, papillary, or diffuse [1, 8–13, 20]. In addition, the immunohistochemical profile of the lung tumor (Fig. 2b–f, Table 1) is different from the bladder tumor, which had a distinctive pattern consistent with CCCB (Fig. 1b–g, Table 1). TTF-1 positivity in the lung tumor further supports a distinct lung primary [21]. Furthermore, genetic testing of the lung cancer demonstrated genomic alterations in *ARID2* and *KEAP1*. Whereas the clinical significance of *KEAP1* is not known, *ARID2*-inactivating mutations have been found in 5% of non-small cell lung cancers and are considered one of the most frequent mutated genes after *TP53*, *KRAS*, *EGFR*, *CDKN2A*, and *STK11* [22]. Unlike pulmonary adenocarcinoma, there is not enough data for targeted therapy of CCCB based on cytogenetic testing. Radical surgery with or without adjuvant radiotherapy (for example, total dose of 50–60 Gy) and/or chemotherapy (for example, cisplatin plus etoposide, doxorubicin, and cyclophosphamide) remains the standard in CCCB management [20].

## Conclusions

Although both tumors in our patient had a similar clear cell component, their different histopathological patterns, secretory properties, and immunohistochemical profiles, along with a unique cytogenetic profile for the lung tumor, favored the presence of two concordant primary tumors composed of clear cells rather than a metastasis from one organ to the other. The concurrence of these two neoplasms is very rare, and more studies are needed to further establish the most appropriate follow-up and management protocols in such challenging cases.

## Abbreviations

AMACR: α-Methylacyl-coenzyme A racemase
CA-125: Cancer antigen 125
CAM5.2: A low-molecular-weight keratin
CCCB: Clear cell carcinoma of the bladder
CEA: Carcinoembryonic antigen
CK: Cytokeratin
MC: Mesonephric carcinoma
ND: Not done
P53: Protein 53
PAS-D: Periodic acid-Schiff with diastase stain
PAX2: Paired box 2
PAX8: Paired box 8
RC: Radical cystectomy
TTF-1: Thyroid transcription factor 1
TURBT: Transurethral resection of bladder tumor
WHO: World Health Organization
